# Apobec-1 Complementation Factor (A1CF) Inhibits Epithelial-Mesenchymal Transition and Migration of Normal Rat Kidney Proximal Tubular Epithelial Cells

**DOI:** 10.3390/ijms17020197

**Published:** 2016-02-02

**Authors:** Liyuan Huang, Honglian Wang, Yuru Zhou, Dongsheng Ni, Yanxia Hu, Yaoshui Long, Jianing Liu, Rui Peng, Li Zhou, Zhicheng Liu, Zhongshi Lyu, Zhaomin Mao, Jin Hao, Yiman Li, Qin Zhou

**Affiliations:** 1 Division of Molecular Nephrology and the Creative Training Center for Undergraduates, the Ministry of Education Key Laboratory of Laboratory Medical Diagnostics, the College of Laboratory Medicine, Chongqing Medical University, Chongqing 400016, China; lyhuang0603@sina.com (L.H.); hackie_wang@126.com (H.W.); zhouyuru93@sina.com (Y.Z.); dongshengni@outlook.com (D.N.); hyx_zuichu@outlook.com (Y.H.); lys960110@sina.com (Ya.L.); keithljn@sina.cn (J.L.); pengrui911@foxmail.com (R.P.); shmily900519@foxmail.com (L.Z.); liuzhicheng323@163.com (Zhi.L.); zhongshilyu@sina.com (Zho.L.); zhaominmao@sina.com (Z.M.); lanyxiu@163.com (J.H.); 18715854036m0@sina.cn (Yi.L.); 2 The College of Laboratory Medicine, Chongqing Medical University, Chongqing 400016, China

**Keywords:** A1CF, EMT, kidney tubular epithelial cells

## Abstract

Apobec-1 complementation factor (A1CF) is a member of the heterogeneous nuclear ribonucleoproteins (hnRNP) family, which participates in site-specific posttranscriptional RNA editing of apolipoprotein B (apoB) transcript. The posttranscriptional editing of apoB mRNA by A1CF in the small intestine is required for lipid absorption. Apart from the intestine, A1CF mRNA is also reported to be highly expressed in the kidneys. However, it is remained unknown about the functions of A1CF in the kidneys. The aim of this paper is to explore the potential functions of A1CF in the kidneys. Our results demonstrated that in C57BL/6 mice A1CF was weakly expressed in embryonic kidneys from E15.5dpc while strongly expressed in mature kidneys after birth, and it mainly existed in the tubules of inner cortex. More importantly, we identified A1CF negatively regulated the process of epithelial-mesenchymal transition (EMT) in kidney tubular epithelial cells. Our results found ectopic expression of A1CF up-regulated the epithelial markers E-cadherin, and down-regulated the mesenchymal markers vimentin and α-smooth muscle actin (α-SMA) in NRK52e cells. In addition, knockdown of A1CF enhanced EMT contrary to the overexpression effect. Notably, the two A1CF variants led to the similar trend in the EMT process. Taken together, these data suggest that A1CF may be an antagonistic factor to the EMT process of kidney tubular epithelial cells.

## 1. Introduction

Apobec-1 complementation factor (A1CF) is an RNA binding protein, containing three RNA recognition motifs (RRMs) in its N-terminus [1]. It has nanomolar affinity to the 11 nucleotide AU-rich region called “mooring” sequence of apolipoprotein B (apoB) mRNA to direct mediate C-to-U RNA editing of apoB mRNA transcript which results in the translational termination of a truncated protein, apoB48 rather than apoB100 [2,3,4]. In this process, A1CF binds to both a cytidine deaminase named apobec-1 and nucleotide C6666 of apoB mRNA for site-specific C to U editing turning CAA to UAA stop codon [5,6]. A1CF has several variants, including A1CF64 and A1CF65, A1CF65 cDNA is different from A1CF64 because of insertion of 24 nucleotides encoding eight amino acids (EIYMNVPV motif) at exon 12, and they support equivalent levels of apoB mRNA editing in cells [7,8]. In order to highlight their construction characters, in this paper, we named the full length variant (A1CF65) as A1CF, and the other one (A1CF64) as A1CF (-8AA). Previous studies have demonstrated that, in addition to the intestine, A1CF is also expressed in the kidneys and liver where neither ApoB mRNA nor apobec-1 is expressed, indicating that A1CF must have other biological functions more than apoB mRNA editing [9]. In this regard, “mooring” sequences have drawn greater attention. A number of mRNAs containing “mooring” sequences were identified, some of which encode proteins participating in cell proliferation and signal transduction. For example, it was confirmed that A1CF could bind to interleukin-6 (IL-6) at its 3′UTR to strengthen the stability of IL-6 [10]. Moreover, germ-line deletion of A1CF led to embryonic lethality and knock-down of A1CF caused apotosis in rat hepatoma cells [11]. Whether A1CF has a biological function during the occurrence and development of kidney disease has been an open question in view of the finding that A1CF is also abundant in the kidneys.

Patients with obesity and diabetes mellitus often suffer from chronic kidney disease (CKD), and the population of these patients grows continually all around the world [12]. CKD has gradually become a major public health and social problem across the world, and draws more and more public attention [13,14]. In almost all progressive CKD, kidney fibrosis has been supposed to be the common final outcome and it has been taken as a major predictor of prognosis and an important determinant of renal insufficiency [15,16]. In this sense, exploring the cellular and molecular mechanisms of kidney fibrosis may offer fresh insights into the progress of new therapeutic strategies for CKD. Emerging evidence has shown that epithelial-mesenchymal transition (EMT) plays a key role in the process of kidney fibrosis [17,18,19]. During kidney fibrosis, one-third of myofibroblasts (the main differentiated or functional cells to renal fibrosis) originate from the tubular epithelial cells which undergo EMT [20]. In this process, epithelial cells that have apical–basal polarity and gap junction undergo multiple biochemical changes. For example, their polarity and junction are lost, and their cytoskeleton gets reorganized [21,22]. In addition, the migratory capacity and invasive ability of individual cell are enhanced, the ability to resist apoptosis is elevated, and the production of extracellular matrix (ECM) components are also greatly increased, thus epithelial cells assume the phenotype of mesenchymal cells [23,24,25]. A hallmark of EMT is the downregulation of epithelial phenotype genes (such as *E-cadherin*), and upregulation of mesenchymal phenotype genes (such as *vimentin* and *α-SMA*) [23]. 

In this study, we demonstrated A1CF was expressed from E15.5d in C57BL/6 *mouse* kidney, and focused on A1CF in *mouse* kidney tubules. Moreover, we found that A1CF negatively regulated the EMT process in normal rat kidney proximal tubular epithelial cells. Thus, our results suggest that A1CF has a wide range of functions in the EMT process, which may provide a new insight of the cellular and molecular mechanisms of kidney fibrosis and provide a potential target gene in kidney fibrosis treatment.

## 2. Results

### 2.1. Apobec-1 Complementation Factor (A1CF) Is Highly Conserved among Species

**Figure 1:**
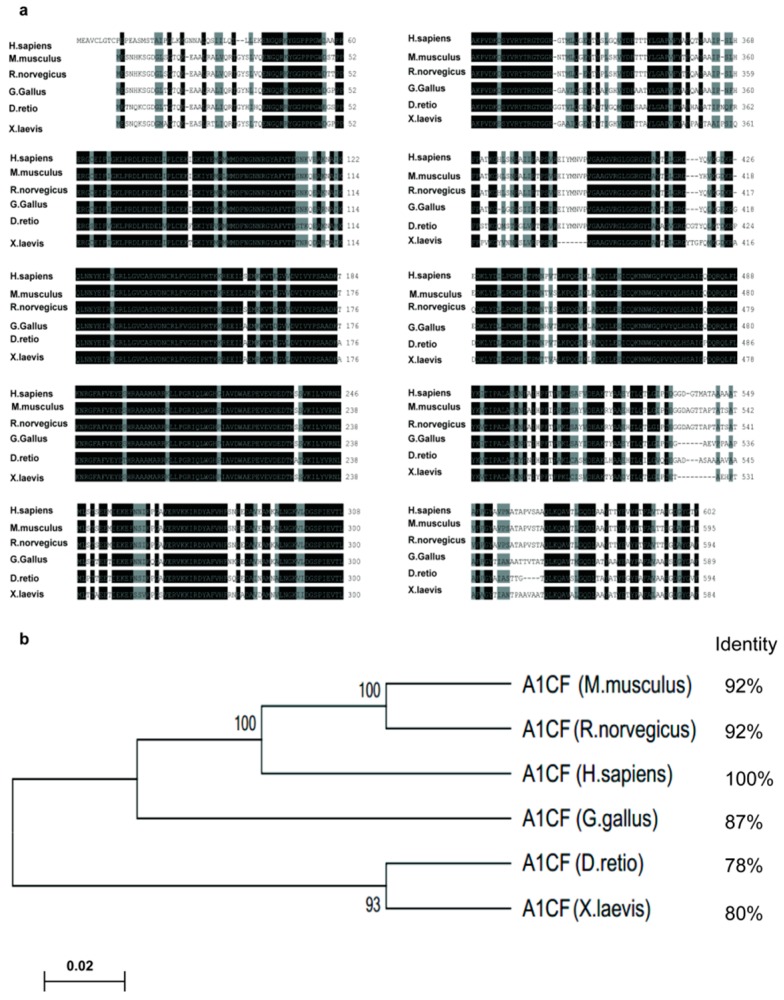
Apobec-1 complementation factor (A1CF) is highly conserved among species. (**a**) A1CF amino acid sequences of *human*, *mouse*, *rat*, *chicken*, *zebrafish*, *xenopus laevis* were selected to analysis. The identical amino acids were shaded in black and the similar amino acids in grey; (**b**) Phylogenetic tree of A1CF amino acids is constructed using MEGA6 software, bootstrap percentages based on 1000 replicates, are shown on each branch. Identity values are listed on the right ranging from 78% to 92%, revealing that A1CF is highly conserved among species.

The transcript variants of A1CF encoding the longest protein sequence were employed to do phylogenetic analysis. The GeneBank accession numbers of these sequences were *H. sapiens* NP_001185748, *M. musculus* NP_001074543, *R. norvegicus* XP_006231334, *G. gallus* NP_421561, *D. rerio* XP_685178, *X. laevis* NP_001086049. DNAssist software was used to compare the amino acid sequences across these species. Results were shown in Figure 1a, the identical amino acids were shaded in black and the similar amino acids were in grey. In order to highlight the conservation of A1CF among species, MEGA6 software was explored to construct the phylogenetic neighbor-joing tree (NJ tree) of A1CF, and NCBI BLAST (http://blast.ncbi.nlm.nih.gov/Blast.cgi) was used to calculate the identity values of these species listing on the right of phylogenetic tree. As shown in Figure 1b, A1CF sequences of *mice* (*M. musculus*), *rats* (*R. norvegicus*), *chicken* (*G. gallus*), *zebrafish* (*D. rerio*), *xenopus laevis* (*X. laevis*) have 78%–92% amino acid identity with *humans* (*H. sapiens*), particularly among *mice* (*M. musculus*), *rats* (*R. norvegicus*), and *humans* (*H. sapiens*), the sequences of *mice* (*M. musculus*) or *rats* (*R. norvegicus*) have 92% amino acid identity with *humans* (*H. sapiens*). Taken together, it is clear to see that A1CF is highly conserved among species, especially among *humans*, *mice* and *rats*.

### 2.2. A1CF Is Highly Expressed in Kidney Tubules

**Figure 2:**
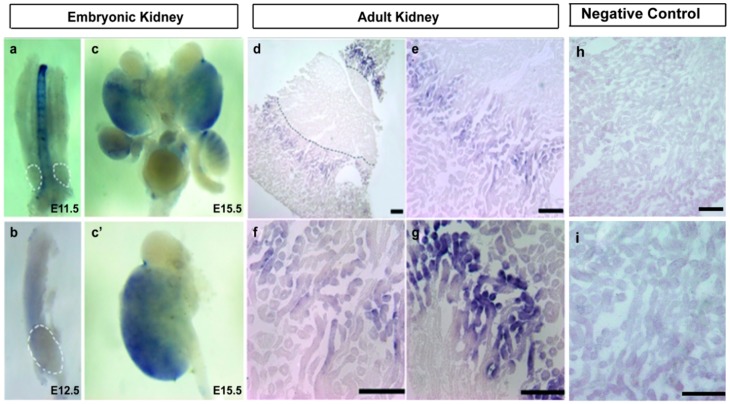
A1CF is expressed in *mouse* kidney starting at E15.5d and centers on the kidney tubules in C57BL/6 *mice*. (**a**–**c**) Whole mount *in situ* hybridization demonstrates that A1CF is expressed in the later stage of kidney development of C57BL/6 *mice*. White dot line indicates the area of metanephros rudiment (original magnification, ×40); (**c′**) To clearly prove the expression of A1CF, metanephros of embryonic day 15.5 (E15.5d) was detected with original magnification, ×100; (**d**–**g**) Section *in situ* hybridization demonstrates that in the adult *mice* kidney, A1CF is mainly expressed in the tubules of inner cortex but not in the other parts of the kidney section; (**d**) The black dot line marks the boundary between cortex and medulla. Scale bar represents 30 µm; (**e**–**g**) Images is the amplification of (**d**). (**e**) Scale bar represents 50 µm; (**f**) Image represents the field surrounding kidney tubules; Scale bar represents 100 µm; (**g**) Image represents the field of kidney tubules. Scale bar represents 100 µm; (**h**,**i**) In control groups no hybridization signal can be detected; (**h**) Scale bar represents 50 µm; (**i**) Scale bar represents 100 µm.

Previous study shows that A1CF is expressed in mammalian kidney [9]. To explore dynamic changes of its expression in kidney development, C57BL/6 *mouse* kidney of embryonic day 11.5 (E11.5d), embryonic day 12.5 (E12.5d) and embryonic day 15.5 (E15.5d) were selected to do whole mount *in situ* hybridization. We also separated adult C57BL/6 *mouse* kidney for section *in situ* hybridization to investigate its tissue localization. Results showed that the expression of A1CF in E11.5d, E12.5d was barely detected (Figure 2a,b). However, in E15.5d, we found that A1CF was weakly expressed (Figure 2c,c′), and the expression of A1CF in adult *mouse* kidneys were obvious (Figure 2d). We also found in the adult kidney of C57BL/6 *mouse*, A1CF was not expressed in the other parts of the kidney section but mainly in the tubules of inner cortex (Figure 2d–g). No hybridization signal can be detected when sense probes were used in control groups (Figure 2h,i). Taken together, our data demonstrated that A1CF is expressed in *mouse* kidneys starting at E15.5d and centers on the kidney tubules in adult *mouse* kidneys.

### 2.3. A1CF Stabilizes Epithelial Character in Rat Kidney Tubular Cells

**Figure 3:**
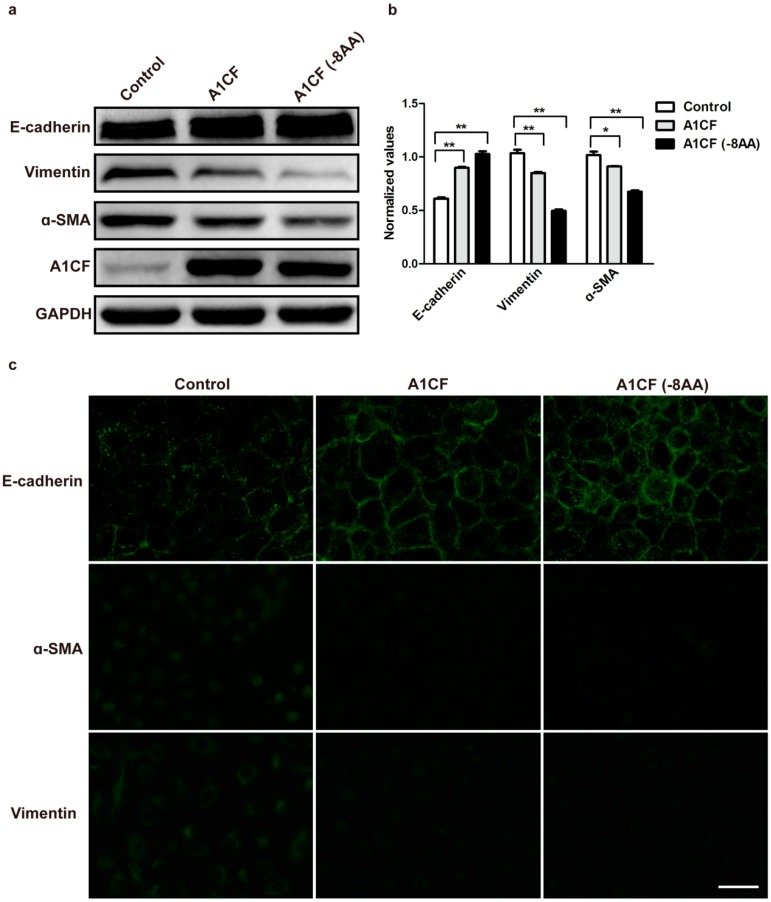
Ectopic expression of A1CF inhibits EMT. (**a**) Overexpression A1CF and A1CF (-8AA) both result in gain of epithelial markers (E-cadherin) and loss of mesenchymal markers (vimentin and α-smooth muscle actin (α-SMA)), and the two variants have the similar trend in this process, GAPDH served as loading control; (**b**) Statistical analysis of relative protein expression levels. Data (mean ± standard error of the mean (S.E.M.)) are representative of three independent experiments. * *p* ≤ 0.05 and ** *p* ≤ 0.01 indicate significant statistical differences compared with the control group; (**c**) Immunofluorescence analysis demonstrates that ectopic expression of A1CF and A1CF (-8AA) potentiate the expression of membrane E-cadherin in NRK52e cells, and the expression of α-SMA and vimentin is weaker compared with control groups (original magnification, ×40). Scale bar represents 50 µm.

**Figure 4:**
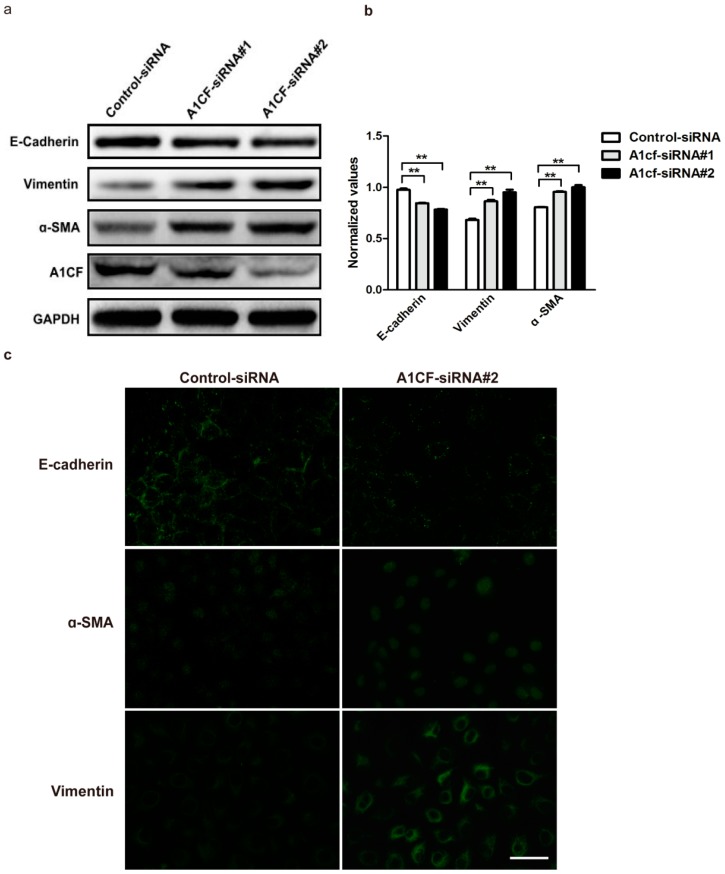
A1CF knockdown induces EMT. (**a**) Control-siRNA, A1CF-siRNA #1, and A1CF-siRNA #2 were transfected into NRK52e cells. Western blotting analysis reveals a decrease in E-cadherin (epithelial marker) and concomitant increase in vimentin and α-SMA (mesenchymal markers). GAPDH served as loading control; (**b**) Statistical analysis of relative protein expression levels*,* Data (mean ± S.E.M.) are representative of three independent experiments. ** *p* ≤ 0.01 indicates highly statistically significant differences compared with control-siRNA group; (**c**) A1CF-siRNA #2 was transfected into NRK52e cells. A1CF knockdown attenuates the expression of membrane E-cadherin and potentiates the expression of α-SMA and vimentin demonstrated by immunofluorescence analysis (original magnification, ×40). Scale bar represents 50 µm.

A1CF amino acids of *mouse* and *rat* were analyzed by NCBI BLAST, and their overlap ratio is 98%, suggesting that the two species are highly homologous. Thus, NRK52e cells that are the immortalized, but non-tumorigenic *rat* tubular epithelial cells were employed to do our experiments. To explore the potential function of A1CF in EMT process, the two variants of A1CF were transiently transfected into NRK52e cells using lipo2000. Epithelial and mesenchymal markers were examined to evaluate the role of A1CF in EMT. As described in Figure 3a,b, the expression of epithelial markers E-cadherin was up-regulated in NRK52e cells, and the expression of mesenchymal markers vimentin and α-SMA was down-regulated. Cells overexpressed with A1CF or A1CF (-8AA) displayed a gain of E-cadherin expression, but a loss of α-SMA and decreased vimentin expression by the immunofluorescence staining (Figure 3c). All the results from Western blot and immunofluorescence staining showed both the two variants of A1CF inhibit EMT in NRK52e cells, more interestingly, we found the variant A1CF (-8AA) deleting EIYMNVPV motif has a stronger effect in this process. In addition, we screened out effective siRNA against A1CF (A1CF-siRNA #1, A1CF-siRNA #2), which could markedly attenuate the expression of A1CF. It was observed that A1CF knockdown led to a decrease of E-cadherin expression but an increase of vimentin and α-SMA expressions (Figure 4a,b). These results were also proved by immunofluorescent assay (Figure 4c). Together, these data suggest that A1CF negatively regulates the process of epithelial-mesenchymal transition (EMT) in *rat* normal kidney tubular epithelial cells.

### 2.4. A1CF Inhibits NRK52e Cells Migration

**Figure 5:**
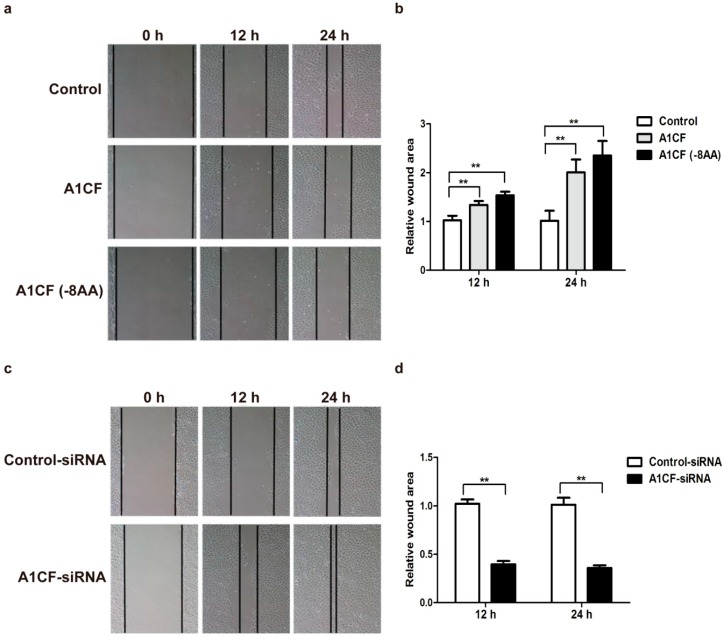
A1CF inhibits NRK52e cells migration. (**a**) Both A1CF and A1CF (-8AA) significantly inhibited NRK52e cells migration. Data were collected at 12 and 24 h (original magnification, ×100); (**c**) A1CF knockdown significantly increases NRK52e cells migration. Data were collected at 12 and 24 h (original magnification, ×100); (**b**,**d**) Data are presented as mean ± S.E.M. of three independent experiments. Statistical analysis was performed using Student’s *t*-test. ** *p* < 0.01 indicated significantly statistical differences compared with control groups.

To further verify the possible correlation of A1CF and EMT. Scratch wound healing assay was employed to investigate the function of A1CF in the process of EMT. NRK52e cells were treated with pCMV-A1CF, pCMV-A1CF (-8AA) and pCMV-flag. As showed in Figure 5a, overexpression of A1CF and A1CF (-8AA) both significantly inhibited the migration ability of NRK52e cells compared to control group. The quantification was analyzed by NIH Image J software, and shown in Figure 5b. In addition, NRK52e cells were also treated by either A1CF-siRNA #2 or Control-siRNA. It was observed that A1CF knockdown remarkably enhanced migration ability of NRK52e cells (Figure 5c). The corresponding quantification was shown in Figure 5d. Thus, our results indicate A1CF inhibits the migration ability of NRK52e cells.

## 3. Discussion

Kidney fibrosis is a remarkable feature of almost all kinds of progressive chronic kidney disease (CKD) [12,26]. In recent years, researchers have demonstrated tubular epithelial cells could undergo transition to a fibroblast phenotype via epithelial-mesenchymal transition [27,28]. Our laboratory is devoted to the research of the molecular mechanism governing kidney fibrosis. In this study, we selected thousands of genes based on the three principles: novel, highly conservative among species and abundant in the kidneys, and A1CF were screened out. In most previous work, A1CF had been limited to function as an auxiliary factor which was required for apoB mRNA editing in mammalian small intestine and liver. However, studies have found that A1CF is also expressed in mammalian kidney where apoB mRNA editing not happens [9], but few studies have involved in this aspect. Thus, it is intriguing to address the role of A1CF in the occurrence and development of mammalian kidney disease.

In this study, we described a novel role of A1CF in the field of mammalian kidneys other than its role in regulating apoB mRNA editing in mammalian small intestines. Our work explored the expression of A1CF along the kidney development phase and the location in mammalian kidneys for the first time. We found that in C57BL/6 mice A1CF is not expressed in early kidney development until E15.5d, and it is abundant in adult mice. Then, we investigated the specific location of A1CF in adult *mouse* kidneys, and our data showed that A1CF was mainly expressed in the tubules of inner cortex. Thus, we hypothesized that A1CF may play an important role in kidney tubules. As demonstrated in our study, A1CF could inhibit the process of EMT in *rat* normal tubular epithelial cells, because ectopic expression of A1CF impaired the process of EMT, and A1CF knockdown enhanced this process in NRK52e cells. We also identified both the two important variants, A1CF64/65, can inhibit the process of EMT according to previous reports that A1CF64/65 supported apobec-1 (the catalytic subunit of the editosome) equivalently in editing of apoB mRNA, suggesting that both A1CF64 and A1CF65 may be act as protective factor for kidney fibrosis. Thus, A1CF might act as a potential molecular target gene for inhibiting kidney fibrosis.

In our study, although the two important variants, A1CF64/65, led to the similar trend in EMT process, the variant A1CF64 deleting the eight amino acids (EIYMNVPV) has a stronger effect in this process, indicating the EIYMNVPV motif may have a potential function. Several years ago, Lellek *et al.* found two different variants of A1CF in *human* tissues, a 586-amino acid protein named A1CF64 and a 594-amino acid protein named A1CF65 [6]. Then numbers of researchers were focused on the two variants. Recent studies have demonstrated that A1CF64 and A1CF65 mRNAs are from the same gene, but A1CF65 cDNA differ from A1CF64 by insertion of 24 nucleotides which encode the eight amino acids (EIYMNVPV). Significantly, the eight amino acids (EIYMNVPV) are identical to intron 11, suggesting that the two proteins are alternatively spliced variants of exon 12. More interestingly, the eight amino acids (EIYMNVPV) are localized on the nuclear localization signal (NLS) domain at the C terminus of A1CF, indicating the two variants may have different subcellular localization. Dance *et al.* has shown that A1CF and A1CF (-8AA) support equivalent levels in the apoB editing events in cells [7]. In this paper, we found that both A1CF and A1CF (-8AA) could inhibit EMT process in NRK52e cells, but A1CF (-8AA) has the stronger effect in this process. However, it remained unclear whether subcellular localization of the two variants or the motif EIYMNVPV itself may lead to their different function, which is worthwhile pursuing in future investigations.

As discussed above, A1CF may have an important role in mammalian kidney, acting as an antagonistic factor of kidney fibrosis by inhibiting the process of EMT. In addition to organ fibrosis, studies demonstrated the pivotal function of EMT in other pathological progressions, including cancer metastasis and wound healing, suggesting that A1CF may have a role during kidney epithelium cancer such as clear cell renal cell carcinoma(ccRCC) and kidney injury healing [24,29,30]. Together, our studies pointed out potent effects of A1CF in mammalian kidney tubular epithelium. However, the precise mechanisms underlying the feature of A1CF and its relation to EMT, and the cellular contexts in which it can contribute to kidney disease remain to be defined.

## 4. Materials and Methods

### 4.1. Cell Culture and Transient Transfection

NRK52e *rat* kidney proximal tubular epithelial cells were cultured in Dulbecco’s modified Eagle’s medium supplemented with 10% fetal bovine serum (FBS, Invitrogen/Gibco, BRL Co., Ltd., Grand Island, NY, USA) and 1% penicillin/streptomycin (Invitrogen, Grand Island, NY, USA), incubated at 37 °C, 5% CO_2_. Lipofectamine 2000 (Invitrogen) was used for plasmids transfections according to the manufacturer’s instructions. In addition, 3 µg of pCMV-flag-A1CF, pCMV-flag-A1CF (-8AA) or vector control pCMV-flag were transfected into NRK52e cells in each well of 6-well plates. Complex was removed after 6 h and then cells were cultured in the corresponding serum-containing media. After 36–48 h, the cells were processed for Western blot or immunofluorescence staining.

### 4.2. Plasmid Construction

A1CF ORF was cloned into the pCMV-flag vector at site of XbaI. Primers: A1CF.F, GGA TCC ACT AGT TCT ATG GAA TCA AAT CAC AAA TCC; A1CF.R, CAC CCG GGA TCC TCT GTT AGA AGG TTC CAT ATG CAT CG. Murine A1CF gene was obtained by PCR, and inserted into pBST vector at site of HidIII and BamHI. Primers: F, TCG ACG GTA TCG ATA ATG GAA TCA AAT CAC AAA TCC; R, GCT CTA GAA CTA GTG AGG TTC CAT ATG CAT CG.

### 4.3. RNA Interference

SiRNA oligonucleotides were purchased from Shanghai GenePharma Co., Ltd. Two stealth siRNAs targeting different sequences of A1CF mRNA were selected to silence A1CF of *Rattus norvegicus*. *Rat*-A1CF-siRNA#2 sense: GCU GCU GCC AGG AAG AAU UTT; and antisense: AAU UCU UCC UGG CAG CAG CTT was the most efficient siRNA for A1CF validated by Western blot (Figure 4). Stealth RNAi negative control, sense: UUC UCC GAA CGU GUC ACG UTT; and antisense: ACG UGA CAC GUU CGG AGA ATT. NRK52e cells were transfected with 100 nM A1CF or control siRNA oligonucleotides using Lipofectamine 2000 (Invitrogen) in 6-well plates. The transfection process was performed according to the manufacturer’s instructions.

### 4.4. Western Blot

Proteins were extracted from NRK52e cells using RIPA lysis buffer (Beyotime, Haimen, China) buffer supplemented with 1 mM phenylmethylsulfonyl fluoride (PMSF) (Beyotime, Haimen, China). Protein concentration was measured by the bicinchoninic acid (BCA) protein assay reagent kit (Merck, Darmstadt, Germany). The protein samples (30 µg each lane) were subjected to 12% SDS-PAGE gels and were electrically transferred to PVDF membrane (Millipore Corporation, Billerica, MA, USA). The blot was blocked by 5% fat-free milk in Tris-buffered saline containing 0.1% Tween-20 (TBST) for 1 h at room temperature, and then was incubated with corresponding primary antibody, such as anti-E-cadherin rabbit pAb (1:1000, Bioworld, Nanjing, China), anti-Vimentin rabbit pAb (1:1000, Bioworld), anti-α-SMA rabbit pAb (1:1000, 14395-1-AP, Proteintech, Chicago, IL, USA), anti-A1CF rabbit pAb (1:1000, ab99955, abcam, Cambridge, MA, USA), or anti-GAPDH rabbit pAb (1:5000, 10494-1-AP, Proteintech), diluted in TBST plus 3% BSA at 4 °C overnight. After washing with TBST, the blots were incubated with horseradish peroxidase (HPR)-conjugated goat anti-rabbit IgG (1:5000, SA00001-2, Proteintech) for 1 h at room temperature. Specific protein bands were visualized by an ECL-plus detection system with chemiluminescent HRP substrate reagent (Millipore Corporation, Billerica, MA, USA). GAPDH was used as a loading control for protein samples.

### 4.5. Immunofluorescence Staining

Cells cultured on clean glass cover slips were fixed with 4% formaldehyde (in 1× PBS), permeabilized with 0.2% Triton X-100 in 1× PBS, and blocked using 5% goat serum (in 1× PBS) for 1 h at room temperature. Then, the cells were stained with anti-E-cadherin Rabbit pAb (1:100, Bioworld); anti-Vimentin Rabbit pAb (1:100, Bioworld); anti-α-SMA Rabbit pAb (1:100, Bioworld) at 4 °C overnight followed by incubating with fluorophore-conjugated goat anti-rabbit IgG-CFL 488 (1:2000, Santa Cruz Biotechnology, Santa Cruz, CA, USA) for 1 h at room temperature. Images were captured using a fluorescence microscope (ECLIPSE Ti-s, Nikon, Tokyo, Japan); and relevant images were taken with a SPOT Diagnostic (Sterling Heights, MI, USA) CCD camera.

### 4.6. Mice and in Situ Hybridization

C57BL/6 mice embryos of E11.5d, E12.5d and E15.5d and adult mice weighing 20–22 g were acquired from the Specific Pathogen-Free Laboratory Animal Center of Chongqing Medical University and maintained according to the guidelines of the Institutional Animal Care at Chongqing Medical University. Animal experiments were done in compliance with protocols (SYXK2012-0001, September 2012) approved by the Ethic Committee of Chongqing Medical University.

We dissected embryonic kidney tissues of C57BL/6 *mouse* of different developmental stages. In our experiment, C57BL/6 *mouse* kidney of E11.5d, E12.5d and E15.5d were selected for whole mount *in situ* hybridization, and we also separated the kidney of adult C57BL/6 *mouse* into section for *in situ* hybridization [31]. The digoxigenin-labeled RNA probe was transcripted from probe vector pBST-m. After linearization with proper restriction endonuclease, digoxin-labeled sense (used as control) RNA probe was transcripted *in vitro* with T7 RNA polymerase (Thermo Scientifics, Waltham, MA, USA), and purified by LiCl/ethanol precipitation. For whole-mount *in situ* hybridization, embryonic kidney or urinogenital ridge at indicated embryonic day were isolated from pregnant C57BL/6 mice and fixed in 4% paraformaldehyde for 24 h at 4 °C. After rinse in PBS, the embryonic kidney and uriogenital ridge were dehydrated into methanol and stored at −20 °C. For section *in situ* hybridization, kidney was harvested from an adult C57BL/6 *mouse* consecutively perfused with PBS and 4% paraformaldehyde and further fixed in 4% paraformaldehyde in small slices for 24 h at 4 °C. After an extensive wash with PBS, the kidney slice was dehydrated with 30% sucrose (in PBS) and embedded in an opti-mum cutting temperature (OCT) compound (Sakura, Japan). Cryo-section was made at 20–30 µm and stored at −80 °C. Whole mount hybridization and section hybridization was preformed as described previously [31]. Images of whole mount *in situ* hybridization were got by stereo microscope (SteREO Discovery.V8, ZEISS, Heidenheim, Germany); and images of section *in situ* hybridization were got by a fluorescence microscope (ECLIPSE Ti-s, Nikon, Tokyo, Japan), and relevant images were taken with a SPOT Diagnostic (Sterling Heights, MI, USA).

### 4.7. Scratch Wound Healing Assay

NRK52e cells were cultured in 6-well plates in Dulbecco’s modified Eagle’s medium (DMEM) supplemented with 10% FBS. When cells confluence reached 50%, pCMV-flag-A1CF, pCMV-flag-A1CF (-8AA) or vector control pCMV-flag were introduced into NRK52e cells. The monolayer cells were scraped using a yellow pipette tip to generate scratch wounds when cells reached 95%–100%, then washed twice with 1× PBS to wipe off cell debris. Cells were incubated in completed DMEM starved serum at 37 °C, 5% CO_2_. Time lapse images were acquired at different time points (12 and 24 h) using a fluorescence microscope (ECLIPSE Ti-s, Nikon, Tokyo, Japan); and relevant images were taken with a SPOT Diagnostic (Sterling Heights, MI, USA) CCD camera. Images were collected from five independent selected fields in each sample, and the wound areas were calculated by NIH Image J software (National Institutes of Health, Bethesda, MD, USA).

### 4.8. Statistical Analysis

All the experiments were performed independently for three times. All graphical values were represented by the mean ± standard error of the mean (S.E.M.). The differences between two groups were analyzed using unpaired two-tailed Student’s *t-*test by GraphPad Prism 5 software (GraphPad Software, Inc., La Jolla, CA, USA). Categorical data were analyzed by Fisher’s exact test. *p*-values of less than 0.05 were considered statistically significant.

## 5. Conclusions

In summary, A1CF is highly conservative among various species by bioinformatics analysis, and it is expressed in a later development phase of *mouse* kidneys rather than the early kidney development stage, and it was mainly expressed in the tubules of inner cortex in adult *mouse* kidneys. We also demonstrated that A1CF could inhibit the process of EMT as ectopic expression or knockdown of A1CF impaired or enhanced EMT in normal *rat* kidney proximal tubular epithelial cells NRK52e cells, respectively, and the two variants, A1CF64/65, led to the similar trend in the EMT process. Thus, A1CF might act as a potential molecular target gene for inhibiting kidney fibrosis.

## Abbreviations

A1CF: Apobec-1 complementation factor
hnRNP: heterogeneous nuclear ribonucleoproteins
apoB: apolipoprotein-B
EMT: epithelial-to-mesenchymal transition
IL-6: interleukin-6
CKDS: chronic kidney diseases
α-SMA: α-smooth muscle cell actin
ECM: extracellular matrix
RRMs: three RNA recognition motifs
NLS: the nuclear localization signal
ccRCC: clear cell renal cell carcinoma
OCT: opti-mum cutting temperature
DMEM: Dulbecco’s modified Eagle’s medium
